# Unicompartmental knee arthroplasty has higher revisions than total knee arthroplasty at long term follow-up: a registry study on 6453 prostheses

**DOI:** 10.1007/s00167-020-06184-1

**Published:** 2020-08-01

**Authors:** A. Di Martino, B. Bordini, F. Barile, C. Ancarani, V. Digennaro, C. Faldini

**Affiliations:** 1 Department of Biomedical and Neuromotor Science – DIBINEM, University of Bologna, Bologna, Italy; 2 1st Orthopaedic and Traumatologic Clinic, IRCCS Istituto Ortopedico Rizzoli, Bologna, Italy; 3 Medical Technology Laboratory, IRCCS Istituto Ortopedico Rizzoli, Bologna, Italy

**Keywords:** Unicompartmental knee arthroplasty, Modes of failure, Revision rate, Usage, Arthroplasty registry, Registry study, Total knee arthroplasty

## Abstract

**Purpose:**

The purpose of this study is to analyse long-term unicompartmental knee arthroplasty (UKA) focusing on survivorship, causes of failure and revision strategy.

**Methods:**

This study is a retrospective analysis of data from a regional arthroplasty registry for cases performed between 2000 and 2017. A total of 6453 UKAs were identified and the following information was analysed: demographic data, diagnosis leading to primary implant, survivorship, complication rate, causes of failure, revision strategies. UKA registry data were compared with total knee arthroplasty (TKA) registry data of 54,012 prostheses, which were implanted in the same time period.

**Results:**

6453 UKAs were included in the study: the vast majority of them (84.4%) were implanted due to primary osteoarthritis followed by deformity (7.1%) and necrosis of the condyle (5.1%). When compared to TKA, UKA showed lower perioperative complication rate (0.3% compared to 0.6%) but higher revision rate (18.2% at 15 years, compared to 6.2% for TKA). No correlation was found between diagnosis leading to primary implant and prosthesis survival. The most frequent cause of failure was total aseptic loosening (37.4%), followed by pain without loosening (19.8%). Of the 620 UKAs requiring revision, 485 were revised with a TKA and 61 of them required a re-revision; on the other hand, of the 35 cases where another UKA was implanted, 16 required a re-revision.

**Conclusion:**

UKA is associated with fewer perioperative complications but higher revision rates when compared to TKA. Its survivorship is not affected by the diagnosis leading to primary implant. Revision surgery of a failed UKA should be performed implanting a TKA, which is associated with a lower re-revision rate when compared to another UKA.

**Level of evidence:**

Level 3, therapeutic study.

## Introduction

Unicompartmental knee arthroplasty (UKA) was introduced in the 1970s as an alternative to total knee arthroplasty (TKA) for single-compartment osteoarthritis (OA) of the knee [24]. The well-known advantages of this type of implant over TKA include bone stock and soft tissue preservation, central pivot retaining, earlier recovery, and better functional outcome [1, 37]; moreover, patients report a subjective feeling of a more natural knee when compared to TKA [36, 37]. Therefore, in the last two decades, there has been a growing interest in the use of UKA implants [13, 37].

Despite the overall encouraging results, UKAs still show a relatively higher revision rate compared to TKA implants [13, 19, 20, 21, 27, 35, 37]. The mechanisms of failure are poorly understood and a consensus on the causes and proper treatment methods has been elusive [20, 24, 35]. Moreover, the type and incidence of perioperative complications depend on the type and design of implants, in the follow-up period, and on the expertise of the surgeon performing the procedure [14, 15].

With the clear potential advantages of UKA, critical evaluation of the correct indications and corresponding causes of UKA failure is necessary. To this effect, only a few studies report the results of large datasets from European National or Regional Registries; for this reason, a registry report from a European country might contribute to fill this gap.

The purpose of the current study is, therefore, to report the long-term results of a large population of unicompartmental knee replacement implants, by analysing the follow-up data of a regional registry from Italy. In particular, the following questions were the aim of the study: is implanting a UKA a safer and less invasive surgery compared to TKA? How long do UKAs survive and why do they fail? Is there a correlation between diagnosis leading to primary implant and failure of the prosthesis? In case of failure, what is the best and most definitive revision strategy?

## Materials and methods

A registry-based population study has been conducted by reporting and analyzing data collected by the Emilia Romagna orthopaedic arthroplasty implants register (called RIPO) [3, 4, 6]. Emilia Romagna (ER) is an Italian region with 4.5 million inhabitants and reports data about hip, knee and shoulder arthroplasty procedures performed inside the region are collected in the register RIPO (Registro Implantologia Protesica Ortopedica). Founded in 1990, RIPO has a capture rate of approximately 95% on the implants performed in all orthopaedic departments of the region (both public and private), involving a total of 62 hospitals. The design of this register was conceived to allow the comparison with the most important national registries.

For the research, knee arthroplasty implants in the period between 2000 and 2017 were considered. Failures were recorded up to the 31/12/2017.

The extraction from the database was made on 21/06/2019. A total of 94,840 primary knee replacement procedures were performed in ER during the selected period, 10,971 of which being femorotibial (medial or lateral) unicompartmental and 83,869 being bi- or tricompartmental implants. No patellofemoral implants were considered.

All procedures performed on patients living outside ER (4518 UKA and 29,857 TKA) were excluded, to minimize bias due to loss to follow-up; in fact, if a patient residing outside ER has primary surgery in this region but revision surgery outside, RIPO does not capture it and survival data would be biased.

**Fig. 1:**
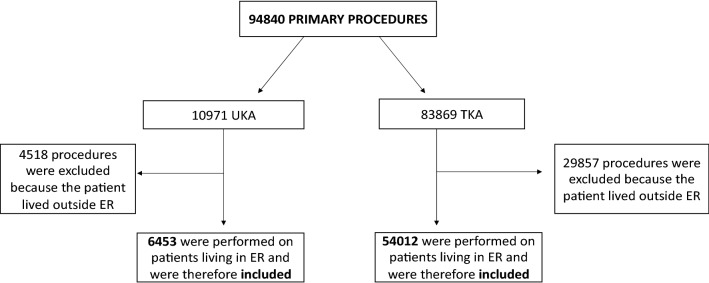
Flowchart showing included and excluded patients and procedures (*ER* Emilia Romagna)

A total of 6453 UKA and 54,012 TKA implants performed on patients living in ER during the 17-year period were included (Fig. 1). The 6453 UKAs were performed on 5,948 patients. About the 5948 included patients receiving a UKA implant, the following information was considered: demographic data, diagnosis leading to primary implant, primary implant survival, perioperative complication rate, number and causes of failure, implant used for revision (TKA or UKA). UKA registry data were compared with total knee arthroplasty (TKA) registry data of 54,012 prostheses, which were implanted in the same time period.

Ethical approval for the study was not necessary because the registry collects data as standard practice on all patients, using a format protecting their identity.

### Statistical analysis

Preoperative features, demographics and causes of revision were presented as percentages of the total cohort. Complication rates were compared using Chi Square analysis. The survival curves were calculated and plotted using Kaplan–Meier method.

Differences between groups were considered as statistically significant for *p* values < 0.05. The Cox multiple regression model was considered to calculate the hazard ratio.

The proportionality hazards assumption was tested by the Schoenfeld residual method; the Wald test was used to calculate the *p* values for data obtained from the Cox multiple regression analyses.

All data came from the registry. The study was a population study including all UKA procedures undertaken on patients living in ER reported by RIPO; therefore, the sample size was not calculated given that all the cases were included.

Revision for any cause was set as the endpoint; revision was defined as the removal or change of any implant that would impact the survival rate.

The statistical analysis was performed using SPSS 14.0 for Windows, version 14.0.1 (SPSS Inc, Chicago, IL, USA) and JMP, Version 12.0.1 (SAS Institute Inc., Cary, NC, 1989–2007).

## Results

The analysis of the population started from the description of demographics and surgical indications. Of the 5948 included patients, 67.9% were females with a mean age of 67 years (range 24–92).

The most common diagnoses leading to primary implant were primary osteoarthritis in the vast majority of patients (84.4%; 5,445 knees), 7.1% deformity and 5.1% necrosis of the condyle, and post-traumatic osteoarthritis (1.9%). Considering implant design, the insert was fixed in 5475 (84.8%), while 972 (15.1%) were mobile bearing; data about the insert were missing in 6 implants (0.1%).

UKA surgery showed lower perioperative complication rate when compared to TKA (Table 1); in fact, both intraoperative and postoperative complications were more frequent in TKA (321/54,012 procedures; 0.6%) compared to UKA (20/6,453 procedures; 0.3%), *p* = 0.004.

**Table 1:** Intraoperative and postoperative complications in UKA and TKA

Complication	UKA	TKA
Intraoperative		
Tibial fracture	5 (0.1%)	24 (0.04%)
Femoral fracture	5 (0.1%)	45 (0.1%)
Anesthesiological complications	1 (0.02%)	16 (0.03%)
Tibial tuberosity or patellar tendon lesions	1 (0.02%)	31 (0.1%)
Collateral ligaments lesions	–	29 (0.1%)
Vascular lesions or haemorragic complications	1 (0.02%)	27 (0.05%)
Others	3 (0.05%)	33 (0.1%)
Total	16 (0.2%)	205 (0.4%)
Postoperative		
Early infection	2 (0.03%)	29 (0.1%)
Deep vein thrombosis	2 (0.03%)	87 (0.2%)
Total	4 (0.1%)	116 (0.2%)

**Fig. 2:**
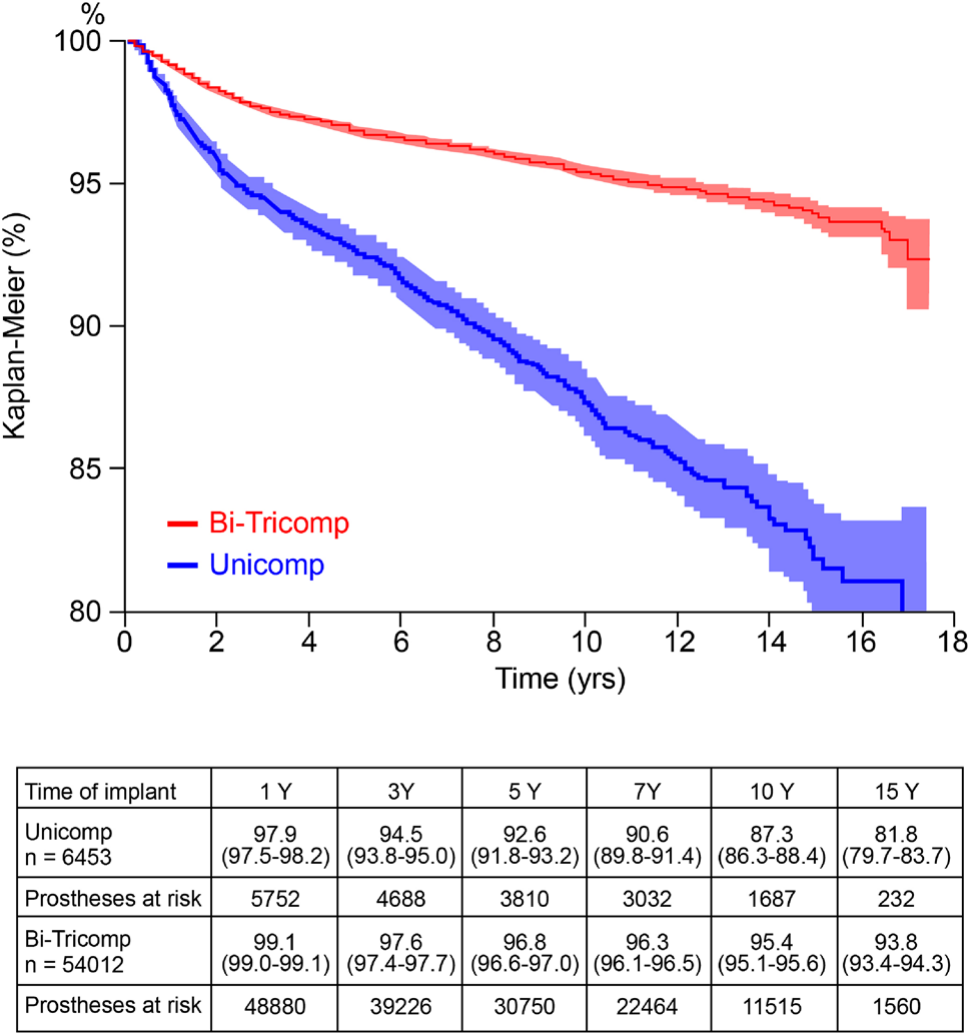
Kaplan–Meier curves showing survivorship of total (red) and unicompartmental (blue) knee replacements. Yrs = years

The revision rate was higher in UKA when compared to TKA. The cumulative survival of UKA implants was 92.6% (CI 91.8–93.2) at 5 years, decreasing to 81.8% (CI 79.7–83.7) at 15 years follow-up; when compared to TKAs, these latter showed a significantly (*p* < 0.05) higher survival at 5- and 15-year follow-up: 96.8 (CI 96.6–97.0) and 93.8% (CI 93.4–94.3), respectively (Fig. 2).

Of the 6453 implants described in this study, 620 failed during the follow-up period; of these, 51.1% failed within the first 3 years after surgery. UKA survival curve (blue line, Fig. 2) showed a quicker decline within the first 3 years after surgery; then, the slope became more gradual.

Cox multivariate analysis showed that the survival unicompartmental knee prosthesis is negatively influenced by type of insert. Mobile insert is worse than fixed insert with a hazard ratio of 1.3 (95% Confidence Interval 1.2–1.4, *p* = 0.0031).

**Fig. 3:**
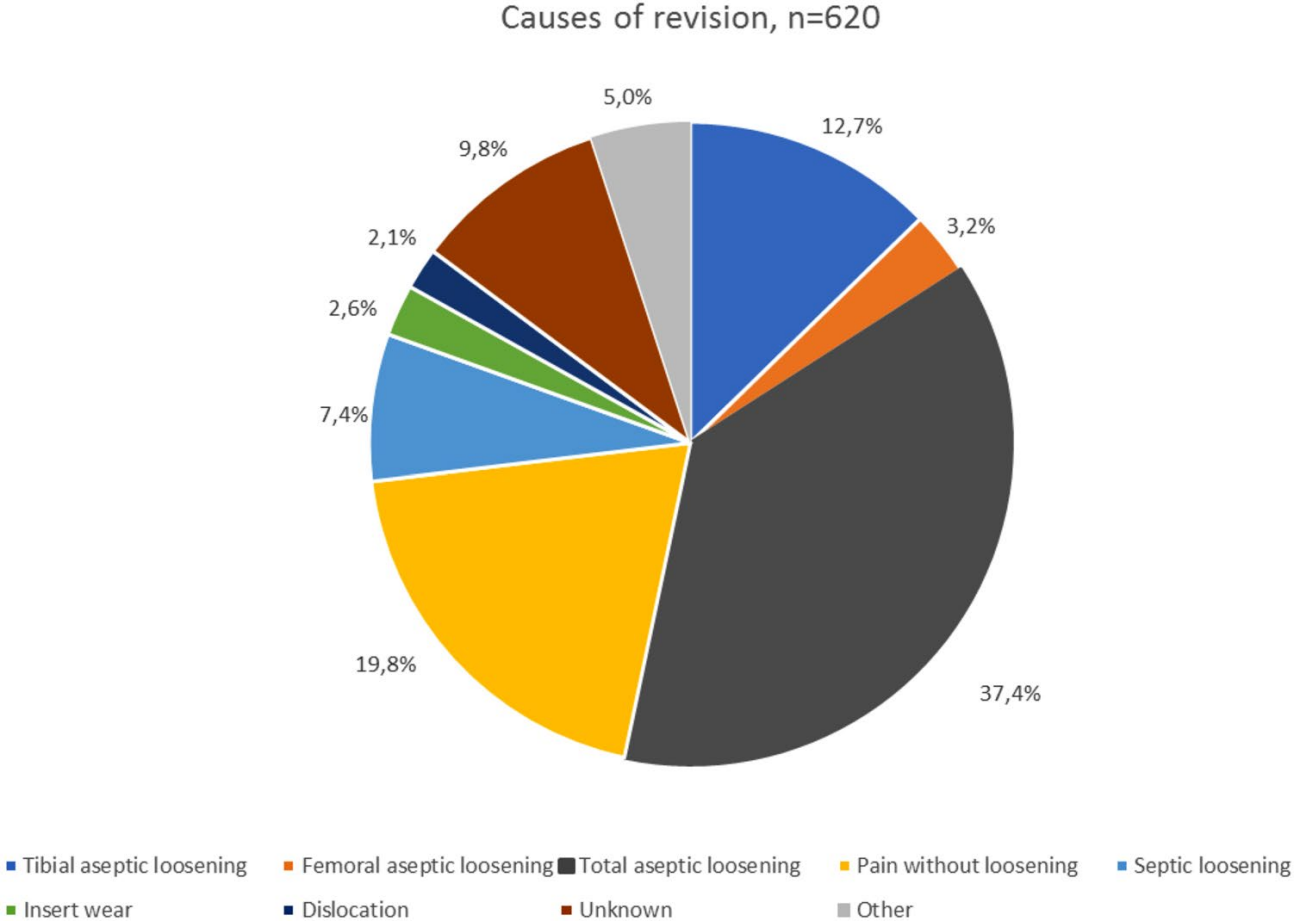
Causes of UKA failure leading to surgical revision

**Fig. 4:**
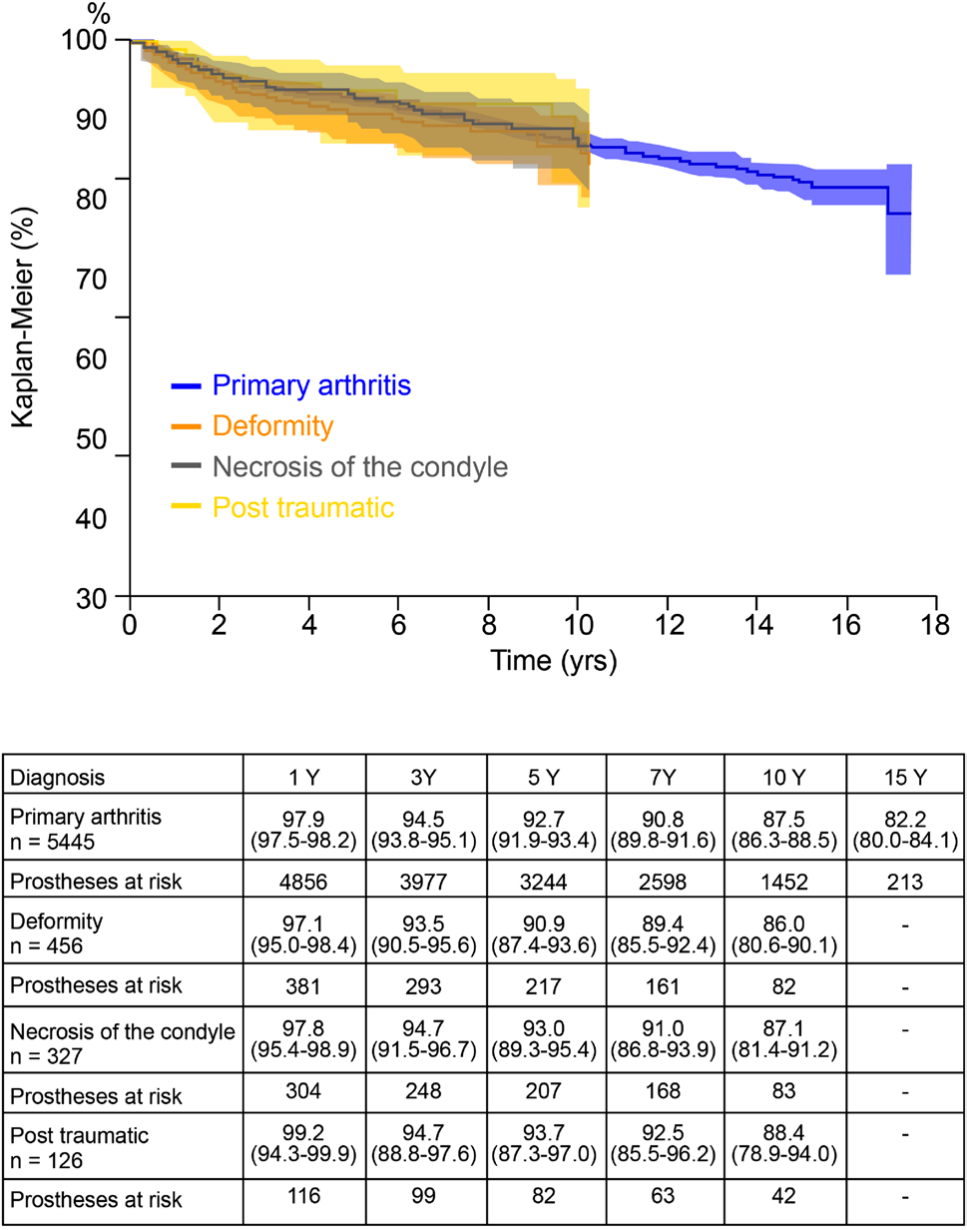
Kaplan–Meier curves of implants survival related to the primary diagnosis leading to UKA. *Yrs* years

Considering the causes of failure (Fig. 3), the most frequent was aseptic loosening (total in 37.4%; only tibial in 12.7%; only femoral in 3.2%). No significant correlation was found between the diagnosis leading to primary implant and implant survival (Fig. 4).

The vast majority of the failed UKAs were revised with a TKA. Of the 620 implants that required revision, 485 were revised with a bi- or tricompartmental TKA, 35 with another UKA, 70 were implant removal and 30 performed outside region. A significantly increased number of re-revisions were performed in UKA (16/35; 45.7%) vs TKA (61/485; 12.5%) (*p* < 0.05).

## Discussion

The most important finding of the present study was that UKAs resulted to be associated with fewer perioperative complications compared to TKA, but also to a higher rate of revisions. Mobile insert UKA showed a significantly increased risk of revision surgery, with a hazard ratio of 1.3. Revision surgery of a failed UKA, if performed by a TKA, is associated with a better implant survival and lower rates revision surgery.

Considering the safety and low invasiveness of UKA procedure, our results lead to the same conclusion reached by multiple authors; coupled with smaller incision surgery and faster recovery protocols, UKA is a less invasive and safer procedure compared to TKA [5, 23, 24, 37]. In particular, we focused on two of the most potentially devastating perioperative complications of any joint replacement (Deep Venous Thrombosis—DVT and periprosthetic infection) and found UKA to have a lower risk of DVT when compared to TKA. Similar results about DVT were obtained by Lombardi et al. [21], who performed a retrospective study regarding the incidence of thromboembolic events after UKA (*n* = 432), and no patient experienced a symptomatic DVT or pulmonary embolism. In addition, Brown et al. [5] compared 2.235 TKAs and 605 UKAs in a retrospective study and reported symptomatic thromboembolic events in 1% of the TKA patients and 0.64% of the UKA patients.

On the other hand, no statistically significant difference was found in periprosthetic infection risk between UKA and TKA group; both groups had the same very low infection rate (0.04%). Higher rates of infection for both UKA (0.3–0.58%) and TKA (0.15–2.5%) implants have been reported [12, 37].

This registry study also compared survival of UKAs to survival of TKAs; an overall UKA implants survival of 81.8% at 15 years was reported. Our results are in agreement with studies from other international registries [16, 28, 29, 32]. In New Zealand, a 15-year implant survival rate of 81.1% was recorded [32]. The data were similar in Australian and UK registries with 78% and 83.1% implant survival at 14 years, which were significantly lower than the TKR survival rates of 92.5% and 94%, respectively [28, 29]. The highest UKA survival rate has been reported by Mohammad and Strikland [25, 26], who analysed more than 8000 Oxford implants; however, their research was biased by the fact that Oxford UKAs can be implanted only by specifically trained orthopaedic surgeons.

Many authors have tried to explain this difference in revision rate and studied the most common causes of UKA failure [28–30, 34, 35]: in a study including 3,967 medial UKA, Van der List et al. reported aseptic loosening (36%), OA progression (20%) and pain (14%) to be the main reasons for failure [33]. Similarly, in our cohort, aseptic loosening was found to be the leading cause (53.38%, including single tibial or femoral component loosening, and total loosening) and pain as the second most common cause of revision (19.8%). Our data are in agreement with the results of other international registries: Australian, UK and New Zealand Registries report loosening (35–40%), progression of disease (20–30%), and pain (8–16%) as the three most important causes of UKA failure [28–30].

A multicentre analysis by Epinette et al. [10] and the UK National Registry [28] both showed that the majority of aseptic loosening cases occurred in the early period (within 2 years postoperatively) and are related to technical pitfalls including malalignment, under or over correction of deformity, mal positioning of components.

Moreover, several studies have shown that high-volume centres and surgeons who performed more UKA per annum had significantly lower revision rates [21, 27, 37]. The New Zealand Registry reported a lower revision rate among surgeons who perform more than 10 UKAs/year (6%) compared to surgeons who perform less than 10 (10%) [32]. This illustrates the importance of the technical aspects in the performance of this surgery, in addition to accurate patient selection. For this reason, improvements in instrumentation and surgical technique, along with assistive devices, such as computer navigation and robotic-assisted surgery, might lead to better accuracy and reduction of technical errors, hopefully improving survivorship of UKA implants [11].

According to our results, insert type has an influence on survival; in particular, mobile-bearing implants are associated with a slightly higher revision rate; this result may have been influenced by the low number of mobile-bearing UKA implanted (15.1%) and the consequently lower surgical experience. In fact, the highest mobile-bearing implants survivorship rates have been reported by high-volume centres [9]. This remains a controversial topic; the bearing modularity continues to incite debates and the superiority of one design over the other has not been demonstrated yet. Other arthroplasty registries who compared fixed and mobile-bearing designs of UKA suggested no conclusive advantage of one over another, citing differing reasons for the failure of each design [17, 31]. In particular, while mobile-bearing implants have a higher re-operation rate due to aseptic loosening and arthritis progression, they show an almost absent incidence of insert wear [8, 33]. In 2015, Ko et al. [15] analysed comparative studies between fixed and mobile bearings focussing on complications: the overall re-operation rate per 100 component years was comparable between the two groups. Nevertheless, the mobile bearings resulted to be more prone to re-operations in patients from aseptic loosening, progression of arthritis and implant dislocation. Therefore, despite the fundamentally different design concepts, both bearings yield similar long-term outcomes and the debate remains open.

Considering surgical indication, no difference was found in survival of implants depending on the diagnosis at primary surgery: our data highlight that UKAs implanted for post-traumatic arthritis, post-traumatic necrosis or deformity did not show a higher risk of failure compared to those implanted for primary arthritis. Only few studies and no other national registry explored this topic. Chalmers et al. [7] found survivorship of UKAs for primary osteonecrosis to be 93% at 10 years, and stated that, as long as correct surgical indication is respected, the diagnosis leading to UKA does not affect implant outcome.

Finally, analysis of revision strategies in the case of UKA failure showed a considerably better outcome when the failed UKA was converted to a TKA (U2T) rather than to another UKA (U2U): 82.6% (CI 77.8–86.5) vs 46.9% (CI 28.0–66.6) at 10 years, *p* < 0.05. These data are in agreement with reports by other national registries, such as the Swedish knee arthroplasty register [17, 18], in which the cumulative rate of re-revision was 7% for U2T and 26% for U2U. The disproportionate number of failed U2U implants suggests that a simple exchange of bearing surfaces does not address the fundamental cause of failure (such as disease progression, malalignment or unequal flexion–extension gap).

We can, therefore, state that revision of a failed UKA with another UKA is associated with an unacceptably high rate of failure and should be avoided when possible.

This study has several limitations. First, it lacks patient-reported outcomes, and information about specific comorbidities associated with inferior arthroplasty survival, such as diabetes or obesity [2, 22, 23], is missing. Second, the registry does not report any functional data about the operated knee. Moreover, the study has all the limitations associated to retrospective studies; as they are not randomized, unmeasured confounders might affect the results. Moreover, the procedures were performed by different surgeons at different hospitals, and results are affected by data collection modality, adherence to the initiative and capture rate.

## Conclusion

UKA is associated with fewer perioperative complications but higher revision rates compared to TKA. Both surgeon and patients should be aware of pros and cons of these implants, which are a good option when lower invasiveness and quick recovery are pursued. In case of failure of a UKA implant, revision surgery by a TKA is associated with a better implant survival and lower requirement of re-revision surgery.

## References

[1] Biazzo A, Masia F, Verde F (2019). Bilateral unicompartmental knee arthroplasty: one stage or two stages?. Musculoskelet Surg..

[2] Bolognesi MP, Marchant MH, Viens NA, Cook C, Pietrobon R, Vail TP (2008). The impact of diabetes on perioperative patient outcomes after total hip and total knee arthroplasty in the United States. J Arthroplasty.

[3] Bordini B, Ancarani C, Fitch DA (2016). Long-term survivorship of a medial-pivot total knee system compared with other cemented designs in an arthroplasty registry. J Orthop Surg Res.

[4] Boyer B, Bordini B, Caputo D, Neri T, Stea S, Toni A (2019). What are the influencing factors on hip and knee arthroplasty survival? Prospective cohort study on 63619 arthroplasties. Orthop Traumatol Surg Res.

[5] Brown NM, Sheth NP, Davis K, Berend ME, Lombardi AV, Berend KR (2012). Total knee arthroplasty has higher postoperative morbidity than unicompartmental knee arthroplasty: a multicenter analysis. J Arthroplasty.

[6] Castagnini F, Sudanese A, Bordini B, Tassinari E, Stea S, Toni A (2017). total knee replacement in young patients: survival and causes of revision in a registry population. J Arthroplasty.

[7] Chalmers BP, Mehrotra KG, Sierra RJ, Pagnano MW, Taunton MJ, Abdel MP (2018). Reliable outcomes and survivorship of unicompartmental knee arthroplasty for isolated compartment osteonecrosis. Bone Joint J..

[8] Cheng T, Chen D, Zhu C, Pan X, Mao X, Guo Y (2013). Fixed- versus mobile-bearing unicondylar knee arthroplasty: are failure modes different?. Knee Surg Sports Traumatol Arthrosc.

[9] Epinette JA, Brunschweiler B, Mertl P, Mole D, Cazenave A (2012). Unicompartmental knee arthroplasty modes of failure: wear is not the main reason for failure: a multicentre study of 418 failed knees. Orthop Traumatol Surg Res.

[10] Gilmour A, MacLean AD, Rowe PJ, Banger MS, Donnelly I, Jones BG (2018). Robotic-arm–assisted vs conventional unicompartmental knee arthroplasty. The 2-year clinical outcomes of a randomized controlled trial. J Arthroplasty..

[11] Hansen EN, Ong KL, Lau E, Kurtz SM, Lonner JH (2019). Unicondylar knee arthroplasty has fewer complications but higher revision rates than total knee arthroplasty in a study of large united states databases. J Arthroplasty.

[12] Johal S, Nakano N, Baxter M, Hujazi I, Pandit H, Khanduja V (2018). Unicompartmental knee arthroplasty: the past, current controversies, and future perspectives. J Knee Surg..

[13] Jones GG, Kotti M, Wiik AV, Collins R, Brevadt MJ, Strachan RK (2016). Gait comparison of unicompartmental and total knee arthroplasties with healthy controls. Bone Joint J..

[14] Kim KT, Lee S, Il Lee J, Kim JW (2016). Analysis and treatment of complications after unicompartmental knee arthroplasty. Knee Surg Relat Res.

[15] Ko Y-B, Gujarathi MR, Oh K-J (2015). Outcome of unicompartmental knee arthroplasty: a systematic review of comparative studies between fixed and mobile bearings focusing on complications. Knee Surg Relat Res..

[16] Koskinen E, Eskelinen A, Paavolainen P, Pulkkinen P, Remes V (2008). Comparison of survival and cost-effectiveness between unicondylar arthroplasty and total knee arthroplasty in patients with primary osteoarthritis: a follow-up study of 50,493 knee replacements from the Finnish Arthroplasty Register. Acta Orthop.

[17] Lewold S, Goodman S, Knutson K, Robertson O, Lidgren L (1995). Oxford meniscal bearing knee versus the Marmor knee in unicompartmental arthroplasty for arthrosis. A Swedish multicenter survival study. J Arthroplasty..

[18] Lewold S, Robertsson O, Knutson K, Lidgren L (1998). Revision of unicompartmental knee arthroplasty: outcome in 1,135 cases from the Swedish Knee Arthroplasty study. Acta Orthop Scand.

[19] Liddle AD, Judge A, Pandit H, Murray DW (2014). Adverse outcomes after total and unicompartmental knee replacement in 101330 matched patients: a study of data from the National Joint Registry for England and Wales. Lancet.

[20] Liddle AD, Pandit H, Judge A, Murray DW (2015). Optimal usage of unicompartmental knee arthroplasty: a study of 41 986 cases from the national joint registry for England and Wales. Bone Joint J..

[21] Lombardi AV, Berend KR, Tucker TL (2007). The incidence and prevention of symptomatic thromboembolic disease following unicompartmental knee arthroplasty. Orthopedics.

[22] Lum ZC, Crawford DA, Lombardi AV, Hurst JM, Morris MJ, Adams JB, Berend KR (2018). Early comparative outcomes of unicompartmental and total knee arthroplasty in severely obese patients. Knee.

[23] Lyons MC, MacDonald SJ, Somerville LE, Naudie DD, McCalden RW (2012). Unicompartmental versus total knee arthroplasty database analysis: is there a winner?. Clin Orthop Relat Res.

[24] Marmor L (1973). Surgical insertion of the modular knee. Rn.

[25] Mohammad HR, Strickland L, Hamilton TW, Murray DW (2018). Long-term outcomes of over 8,000 medial Oxford Phase 3 Unicompartmental Knees—a systematic review. Acta Orthop.

[26] Murray DW, Parkinson RW (2018) Usage of unicompartmental knee arthroplasty. Bone Joint J. 100-B(4):432-435 10.1302/0301-620X.100B4.BJJ-2017-0716.R1 29629577

[27] Nettrour JF, Ellis RT, Hansen BJ, Keeney JA (2020). High failure rates for unicompartmental knee arthroplasty in morbidly obese patients: a two-year minimum follow-up study. J Arthroplasty.

[28] National Joint Registry for England, Wales, Northern Ireland and the Isle of Man (2018) 15th annual report. https://www.hqip.org.uk/wp-content/uploads/2018/11/NJR-15th-Annual-Report-2018.pdf. Accessed 5 June 2019

[29] National Joint Replacement Registry for Australia (2018) Annual report. https://aoanjrr.sahmri.com/documents/10180/576950/Hip%2C%20Knee%20%26%20Shoulder%20Arthroplasty. Accessed 3 June 2019

[30] Robertsson O, Lidgren L (2008). The short-term results of 3 common UKA implants during different periods in Sweden. J Arthroplasty.

[31] Smith TO, Hing CB, Davies L, Donell ST (2009). Fixed versus mobile bearing unicompartmental knee replacement: a meta-analysis. Orthop Traumatol Surg Res.

[32] The New Zealand Joint Registry (2016) Eighteen year report January 1999 to December 2016. https://nzoa.org.nz/system/files/DH7827_NZJR_2017_Report_v4_26Oct17.pdf. Accessed 1 June 2019

[33] van Der List JP, Zuiderbaan HA, Pearle AD (2016). Why do medial unicompartmental knee arthroplasties fail today?. J Arthroplasty.

[34] Vasso M, Corona K, D’Apolito R, Mazzitelli G, Panni AS (2017). Unicompartmental knee arthroplasty: modes of failure and conversion to total knee arthroplasty. Joints.

[35] Waldstein W, Kolbitsch P, Koller U, Boettner F, Windhager R (2017). Sport and physical activity following unicompartmental knee arthroplasty: a systematic review. Knee Surg Sports Traumatol Arthrosc.

[36] Wiik AV, Nathwani D, Akhtar A, Al-Obaidi B, Strachan R, Cobb JP (2019). The unicompartmental knee is the preferred side in individuals with both a unicompartmental and total knee arthroplasty. Knee Surg Sports Traumatol Arthrosc.

[37] Wilson HA, Middleton R, Abram SGF, Smith S, Alvand A, Jackson WF (2019). Patient relevant outcomes of unicompartmental versus total knee replacement: systematic review and meta-analysis. BMJ.

